# Evaluating the impact of a family violence transformational change project in a major trauma hospital: A three-year follow-up comparison study of knowledge, confidence, and family violence response skills in clinical staff

**DOI:** 10.3389/frhs.2022.1016673

**Published:** 2023-01-06

**Authors:** Caroline A. Fisher, Kirsty Troy, Catherine Rushan, Kim Felmingham, Toni D. Withiel

**Affiliations:** ^1^ Allied Health, Family Safety Team, Royal Melbourne Hospital, Melbourne, VIC, Australia; ^2^ Allied Health, Psychology, Royal Melbourne Hospital, Melbourne, VIC, Australia; ^3^ Neuropsychology Service, The Melbourne Clinic, Melbourne, VIC, Australia; ^4^ Clinical Psychology, Melbourne School of Psychological Sciences, The University of Melbourne, Parkville, VIC, Australia

**Keywords:** domestic violence, family violence, hospital, healthcare, intimate partner violence, screening, training

## Abstract

Family violence is a significant public health issue. Healthcare systems have an important role to play in recognising and responding to current family violence experiences in their patients. However, many healthcare workers and systems remain underprepared to fulfil this role. The current study evaluated the impact of a transformational change project in family violence clinical response at a major adult trauma hospital in Australia. Clinician self-rated knowledge, confidence, and family violence clinical skills were evaluated at three years post implementation of a family violence initiative at the Royal Melbourne Hospital, Melbourne. The three years post survey results (*N* = 526) were compared to baseline (*N* = 534) using Mann Whitney *U* and *χ*^2^ analyses. Self-reported clinician family violence knowledge, confidence and patient screening were all significantly improved from baseline. Specific family violence skills, including knowledge of key indicators, enquiry with patients and disclosure response were also all significantly improved. The most common clinician identified barriers to working effectively in the area were similar to baseline and included the presence of a suspected perpetrator during the clinical interaction, clinicians perceiving patients would be reluctant to disclose, and time limitations. However, significantly fewer staff endorsed a lack of knowledge or supporting policies and procedures as a barrier. The findings indicate that investment in a transformational change project comprised of the establishment of response policies and clinical work-flow, broad-scale training, a clinical champions program, a secondary consultation service and links with partner organisations, was effective at improving clinician self-rated rated family violence skills, across the hospital. However, one quarter of clinicians still reported having not received any family violence training, and half endorsed having little or no confidence in their skills to identify and respond to patient family violence experiences. This indicates ongoing and sustained work is required to optimise clinician skills in responding to family violence.

## Introduction

Family violence is behaviour perpetrated by someone in a kinship structure that causes fear and/or physical, emotional, psychological or financial harm (1). This includes intimate partner violence, domestic violence, interpersonal violence, neglect, elder abuse, and child abuse. It is predominantly perpetrated by men against women and children, but can affect people of any gender, sexual orientation and age (2). Globally, violence by a male intimate partner is the most prevalent form of violence against women (3). In Australia, family violence disproportionately affects first nations people, women with disabilities, people from the LGBTIQA+ community, those from culturally diverse linguistic backgrounds and older people (2, 4, 5).

Violence in an intimate partner context is the highest contributing risk factor to disease burden (death, disability, illness) in women aged 25–44 years in Australia (6). In many parts of the world, the wide-spread and devastating impact of family violence has been highlighted through a series of comprehensive reports. In Australia commissions by state governments include the *Not Now, Not Ever* report in Queensland (7), and the *Royal Commission into Family Violence in Victoria* (8). Similar work has been undertaken internationally in Spain, South Africa, the United Kingdom, and the United States (9–12).

The reports generated from these investigations all highlight the importance health systems play in identifying and assisting victim/survivors. This includes healthcare workers implementing either universal or targeted family violence screening and providing support and care planning in situations of moderate and high risk (7–10, 12). The reports also highlight the devastating health and well-being consequences of family violence on victim survivors (7–10). Previous research has indicated that 70% of women killed through family violence utilised medical healthcare services in the 12 months prior to their death, and 25% mental healthcare, whilst very few (3%) sought help from family violence specific services (13). This underscores the importance of healthcare services assisting in family violence situations.

While many communities acknowledge the role healthcare services have in addressing family violence, what is less clear is the effectiveness of service level reforms implemented to address these needs. The evaluation of several pilot trials introducing new family violence screening and support programs in healthcare services have recently been published. A trial of a nurse-delivered intervention addressing intimate partner violence in government-led community health clinics in Mexico found reductions in violence for both female service users that received family violence screening, health/safety risk assessments and supportive referrals, and those that receive a less comprehensive service of family violence screening and referral cards (14). Qualitative research has also been published investigating the experiences of clinical staff participating in the Assessing for Domestic Violence in Sexual Health Environments (ADVISE) trial (15). This indicated that staff trained in the identification and referral to improve safety program went on to prioritise enquiring about family violence in their practice, adapted enquiry to the characteristics of patients, and were comfortable with providing quick on-referrals in low-risk cases. However, challenges were reported working through time-consuming high-risk cases, with modifications to training, regular updates and more resourcing recommended.

How healthcare staff are trained to improve their family violence clinical response is also important to consider. Where this has been evaluated, the results indicate mixed outcomes (16–18). Evaluation of an intervention in a maternal and sexual health service in the United Kingdom resulted in improved knowledge and practice in the short-term, however harms were reported to have occurred during the evaluation period (16). This intervention included implementing guidelines, clinician education, a framework for routine enquiry with all patients, and on-referral to an advocacy service. Despite improved clinician knowledge, issues including failure to document, negative labelling and stereotyping from staff, breaches of confidentiality, and failing to preventing situations of risk including discharging a mother a baby home with an abusive partner, where indicated. In another study, a system's change model was evaluated that utilised a team training approach across several hospitals in the United States (17). In this study, no increase was found in the rates of identification of patients experiencing domestic violence in the emergency departments of participating hospitals; however, improved patient satisfaction and significant and sustained culture change in the emergency departments was indicated.

Both establishing the learning framework, and evaluating the outcomes, in family violence healthcare clinician training scenarios is not straight-forward. The majority of the studies reported above used multiple methods and modalities to effect change within the services, employing broad transformational change or systems change approach to tackle the problem. Thus, success can be determined by looking at any number of broad and specific factors, including clinician knowledge levels, patient screening rates, patient satisfaction, uptake of on-referrals and patient outcomes. Generally, most approaches fall into the framework of cognitivism based instrumental learning theory approaches, with some elements of experiential, transformative and social learning principles utilised as well (19).

In Victoria, Australia, the Strengthening Hospital Responses to Family Violence (SHRFV) initiative was launched by the state government to directly implement Royal Commission recommendations for public health services to provide a whole-of-hospital response to family violence (8). This included three to five years of grant funding for all state health services to implement a transformational change project to improve their family violence response. Several baseline studies have been published from hospitals within this program about staff family violence knowledge and patient perceptions of screening, as well as pilot research on a family violence clinical champions program (20–26). This research indicated that there was general under preparedness to respond to family violence and a wide range of knowledge and skill levels in healthcare clinicians across services and disciplines prior to the implementation of SHRFV. Similarly, the rate at which clinicians screened patients for family violence across services was also inconsistent (20, 23, 26). However, when patients disclosed family violence concerns, the majority indicated that they were happy with the support they received from clinicians (22, 26). The research further indicated some promise for healthcare worker family violence clinical champions programs (24, 25). Evaluations with clinicians in an adult trauma hospital who had participated in a clinical champions program found significant and sustained improvements in self-reported family violence knowledge and skills in both allied health and nursing clinicians. However, knowledge levels and engagement with the initiative were stronger over time in allied health clinicians (24, 25).

In addition to selected SHRFV sites conducting baseline research, an audit tool was recently developed by the Royal Women's Hospital, in conjunction with the University of Melbourne. The tool was designed for whole-of-system evaluation to assess the impact of the SHRFV program at individual hospitals (27). Eighteen SHRFV health services participated in piloting the tool, between November 2019 and 2021 (occurring two to three years after the launch of SHRFV at the services). The tool ranked services across a range of domains and the report from this pilot research suggests that the SHRFV program improved the ability of the services to identify and respond to family violence. However, improvements in patient facing aspects of the program were recommended.

The baseline studies and audit tool report provide useful information; however, to date, no research containing both pre and post SHRFV implementation data to evaluate the effectiveness of the SHRFV program at a healthcare service has been provided. The current study attempts to address this gap by providing a comparison analysis of staff family violence knowledge and clinical skills in a large tertiary adult trauma hospital prior to the implementation of the SHRFV informed transformational change project (baseline), and at 3-years post implementation (follow-up). It is the first study, to our knowledge, to comprehensively evaluate the impact of the SHRFV initiative with both baseline and follow-up measures in a large clinical staff cohort (with *N* = 500+ participating in the survey research in both phases).

This study aimed to evaluate the impact of a transformational change project in family violence clinical response at a major trauma hospital in Melbourne, Australia. The study assessed clinician self-rated knowledge, confidence, and family violence clinical skills three-years following the implementation of a family violence initiative at the Royal Melbourne Hospital.

The paper presents data collected in November-December 2020, from a whole of hospital clinician survey at Royal Melbourne Hospital and compares the survey results to those obtained at baseline in the same health service in November-December 2017 (21), prior to the implementation of service-wide clinician training in family violence.

The Royal Melbourne Hospital was awarded state government grant funding under the SHRFV initiative (described above). Prior to the commencement of the project, the Royal Melbourne Hospital had no family violence clinical response policy or procedure to guide staff when assisting patients experiencing family violence. There was also no internal, hospital-provided, training in family violence clinical response, and no standardised method for screening patients or responding to disclosures. Further, there was no way of tracking, or evaluating, the number of clients presenting to the hospital due to family violence injuries or trauma, or those experiencing family violence being treated at the hospital. Evaluation of the baseline, pre-initiative environment, in regard to clinician family violence skills, patient experiences of family violence screening and clinician responses has been documented in previous studies (21–23, 28).

Details of the Royal Melbourne Hospital design and implementation of the transformational change project to address these issues are presented in Fisher et al (2022) (29). To summarise, this included the development a family violence clinical response policy in 2018, establishment of a specialist multidisciplinary family violence team (the Family Safety Team) to provide training in family violence to both clinical and non-clinical staff hospital wide, and the introduction of a secondary consultation service for all clinicians assisting patients experiencing family violence. Standardised family violence screening and response workflow was also built into the hospital's new electronic medical record (rolled out across the service in 2019 and 2020). This included best practice guideline “pop-ups” to aid clinicians when screening patients for family violence concerns and supporting them following disclosures. Links were made with family violence community service partners and police to facilitate safe patient discharge and care-planning after leaving hospital. A family violence support program was established for staff experiencing family violence, and a family violence research and evaluation program was embedded. Data for the current study were collected at the 3-year follow-up time point when there had been 5,398 staff attendances at family violence training provided by, or sourced through, the Family Safety Team, over the preceding three years. This included the training of 232 Family Safety Advocates clinical champion who had received a minimum of 9 h training and were supported by a community of practice (24, 25).

## Materials and methods

The setting was a large, Tier 1, adult trauma hospital in Melbourne, Australia. The survey methodology was the same as in the baseline study [data collected in 2017], (21), except for some minor adjustments to the survey tool, detailed below. The available work email addresses of all clinical staff were collated (Nursing = 1,829; Medical = 660; Allied Health = 549), and an invitation/reminder to participate in the online survey was sent to staff a maximum of three times over 4 weeks. The survey was open for a total of six weeks. Consent was implied on participation as approved by the Royal Melbourne Hospital Human Research Ethics Committee. The Melbourne Health Human Research Ethics Committee was the approving body: HREC Reference Number: HREC/17/MH/283; SSA Reference Number: SSA/17/MH/390; Research Title: Assisting Patients/Clients Experiencing Family Violence: Clinician Survey.

### Survey tool

The Assisting Patients/Clients Experiencing Family Violence: Royal Melbourne Hospital Clinician Survey was used (21). It is an 11-question survey tool enquiring about the knowledge, the confidence of clinical skills of clinicians in the area of FV. It also surveys clinician endorsed barriers to addressing DFV. The survey consists of Likert-type ordinal responses, forced choice categorical responses (Yes, No, Somewhat) and qualitative free-text response sections (please see (21) Designed for the Victorian context, this survey has been used in three previous studies (with combined clinician participants of *N* = 754) (20, 21, 30). It has good internal consistency, as indicated in previous studies (Withiel et al., 2021, Cronbach's Alpha −0.83; Fisher et al., 2022, Cronbach's alpha of 0.77). It is also capable of differentiating between professions with higher levels of FV training and experience, and is sensitive to changes in knowledge following training (24). Minor modifications were made to the survey at follow-up. Specifically, sections on respondent gender identity and age were added to allow for greater demographic characterisation of the sample. Self-report of prior family violence training was also modified to indicate the specific type(s) of Royal Melbourne Hospital training respondents had attended since the initiative commenced. Finally, a question asking clinicians to estimate their total number of family violence training hours was added (23, 30).

### Analysis

Descriptive statistics are provided for all demographic data. Due to the cross-sectional nature of data collection, changes in ordinal outcomes between baseline and follow up were analysed using a series of Mann Whitney *U* analyses. Differences in nominal outcomes were analysed using a series of *χ*^2^ analyses. A two-tailed alpha of 0.05 was set for the determination of statistical significance. The free-text data obtained in the survey has also been analysed and will be presented in a subsequent paper using qualitative thematic analysis (31).

## Results

A total of 526 clinicians completed the survey at the 3-year follow-up 2020 data collection phase. This was compared to the data provided by 534 participants in the baseline 2017 phase. The nature of the ethics approval obtained for the study (anonymous, not identifiable data collection) did not allow the research team to track clinicians that had participated at both baseline and 3-year follow-up. However, it is likely that some respondents participated in the survey at both time points. Characteristics and demographics are provided in Table 1. Similar to the baseline cohort, almost half the sample had worked in their clinical profession for 10 years or more. More than three quarters identified as having a female gender identity, and 30–39 was the most common age bracket. As with the baseline study, the strongest response rate was seen from allied health clinicians, although unlike baseline, allied health also made up the highest number of respondents, with more participants than nursing and medical clinicians. More clinicians had participated in prior family violence training in the follow-up cohort, and considerably more had participated in training in the past two years. Overall, 48.67% of the follow-up sample endorsed completing at least one specific type of family violence training provided at the Royal Melbourne Hospital since the transformational change project began. Just over a quarter had completed one form of training (27.57%), 16.53% had completed two forms of training, and 4.56% three forms or more. A total of 94 respondents had completed the Royal Melbourne Hospital's Family Safety Advocate training (clinical champions program), providing 9 h+ training (for further information about the types of training provided see (24, 29).

**Table 1:** Sample demographics across baseline and 3-year follow-up.

	Baseline Nov–Dec 2017 (Fisher, Rudkin et al., 2020 and Withiel et al., 2021)	3-Year follow-up Nov–Dec 2020 (current study)
Total sample *N*	534	526
Profession/Subgroup *N* (% of total)		
Nursing	242 (45.32)	213 (40.49)
Acute	141 (26.40)	137 (26.05)
Emergency department	64 (11.99)	44 (8.37)
Subacute	30 (5.62)	22 (4.18)
Other	7 (1.31)	10 (1.90)
Allied health	225 (42.14)	245 (46.58)
Physiotherapy	52 (9.74)	53 (10.08)
Social work	42 (7.87)	41 (7.79)
Occupational therapy	37 (6.93)	40 (7.60)
Clinical Nutrition/ Dietetics	18 (3.37)	18 (3.42)
Speech Pathology/ Audiology	18 (3.37)	20 (3.80)
Psychology	17 (3.18)	17 (3.23)
Other	41 (7.68)	56 (10.65)
Medical	67 (12.55)	68 (12.93)
Acute	24 (4.49)	32 (6.08)
Emergency department	18 (3.37)	10 (1.90)
Subacute	1 (0.19)	8 (1.52)
Outpatients	4 (0.75)	8 (1.52)
Rehabilitation	11 (2.06)	4 (0.76)
Other	9 (1.69)	6 (1.14)
Response rate %		
Overall	17.62	17.1
Nursing	15.70	11.65
Allied Health	53.32	44.63
Medical	6.28	10.30
Years of experience in profession *N* (%)		
<1 year	21 (3.93)	29 (5.51)
1–5 years	136 (25.47)	136 (25.86)
6–10 years	121 (22.66)	122 (23.19)
>10 years	256 (47.98)	239 (45.44)
Age bracket		
<25	NA	23 (4.37)
25–29	NA	101 (19.20)
30–39	NA	186 (35.36)
40–49	NA	122 (23.19)
50–59	NA	65 (12.36)
60–64	NA	23 (4.37)
65+	NA	6 (1.14)
Gender identity %		
Female	NA	414 (78.71)
Male	NA	102 (19.39)
Non-binary	NA	3 (0.57)
Transgender	NA	–
Different identity	NA	–
Prefer not to say	NA	7 (1.33)
Prior family violence training		
None:Some %	35 : 65	25 : 75
Last 2 years - None:Some %	72 : 28	39 : 61
Mean hours	NA	4.31
Standard deviation hours	NA	7.37
Range hours	NA	0–60
Mean hours summed training type category averages^a^	5.90	NA

NA, not available.

^a^ Participants were not asked to provide total number of family violence training hours in the baseline survey. Thus, the average hours of participants endorsements in Table 2 (Fisher, et al., 2020) was taken and summed across all training types to provide an approximate equivalence of total training hours. Unlike follow-up, this also included self-taught learning.

Mann Whitney *U* analyses revealed statistically significant improvement in clinician ratings of their family violence knowledge, confidence and screening rates, and frequency of working clients with family violence experiences. See Table 2 for the comparison of results and statistical significance levels for specific questions. Comparatively, just 23.96% of clinicians rated their family violence knowledge level as *Moderate* or above, at baseline, while at follow-up this had increased to 55.7%. Similarly, those rating their confidence working in the area of family violence as *Moderate* or above, stood at 27.71 percent at baseline, compared to 49.82% at follow-up. Clinicians who had completed training more recently provided stronger confidence ratings (Confidence mean rank: >2 years ago training −126.35, ≤2 year ago training −219.88; Mann Whitney *U* = 18,695.50, *p* < 0.001). However, this is likely to have also been mediated by training amount, with clinicians who had completed training more recently also endorsing a significantly higher number of training hours [Mean (SD) training hours: >2 years ago training −2.97 (9.54); ≤2 years ago training −6.14 (7.46); Independent samples *t*-test, *t*(400) = −3.19009, *p* = 0.002]. For screening, those rating their frequency of screening at *Sometimes*, *Often* or *Always*, increased from 31.05% to 50.00%. However, clinician ratings of their frequency of working with patients experiencing family violence, was not significantly different across the two time points.

**Table 2:** Comparison of clinician's self-ratings of skills and experience in the area of family violence by survey question and survey time-point.

Question	Clinicians ratings as a percentage of the total sample	Test statistic (*p* value)
**FV knowledge rating**	**No knowledge**	**Little knowledge**	**Moderate knowledge**	**Strong knowledge**	**Very knowledgeable**	** **
Baseline	17.79	58.24	18.16	4.68	1.12	
Follow-up	5.51	38.78	40.87	12.55	2.28	*U* = 90,292.00 (<0.001)
**FV confidence rating**	**Not at all confident**	**A little amount confident**	**Moderately confident**	**Confident**	**Very confident**	** **
Baseline	37.27	35.02	19.85	6.55	1.31	
Follow-up	15.21	34.98	31.75	15.60	2.47	*U* = 97,115.50 (<0.001)
**FV screening rate**	**Never**	**Rarely**	**Sometimes**	**Often**	**Always**	** **
Baseline	37.64	31.27	20.00	9.18	1.87	
Follow-up	20.91	29.28	26.43	17.49	6.08	*U *= 105,074.50 (<0.001)
**Frequency of working with patients with FV experiences**	**Never**	**Very seldom**	**Sometimes**	**Often**	**Most of the time/Always**	** **
Baseline	17.19	58.24	18.16	4.68	1.31	*U* = 138,744.50 (0.72)
Follow-up	13.50	39.54	36.69	8.56	1.71	

Similar to the baseline data set, secondary analysis of the 3-year follow-up survey results revealed differences in mean rankings according to profession grouping (see Table 3 for results and Kruskal-Wallis analysis). Allied health clinicians self-rated knowledge and confidence levels were higher than medical and nursing clinicians. However, medical staff endorsed working with patients who had experienced family violence more frequently than the other profession groups.

**Table 3:** Mean ranks for self-reported family violence skills between professional groups.

Family violence skill area	Nursing	Medical	Allied health	Test statistic (*p* value)
Knowledge	239.58	229.34	293.78	*H* = 21.16 (<0.001)
Confidence	249.34	234.51	283.86	*H* = 9.50 (0.009)
Frequency of screening	271.67	275.71	253.01	*H* = 2.36 (0.31)
Frequency of working with patients experiencing violence	250.37	315.88	260.38	*H = *11.03 (0.004)

**Figure 1:**
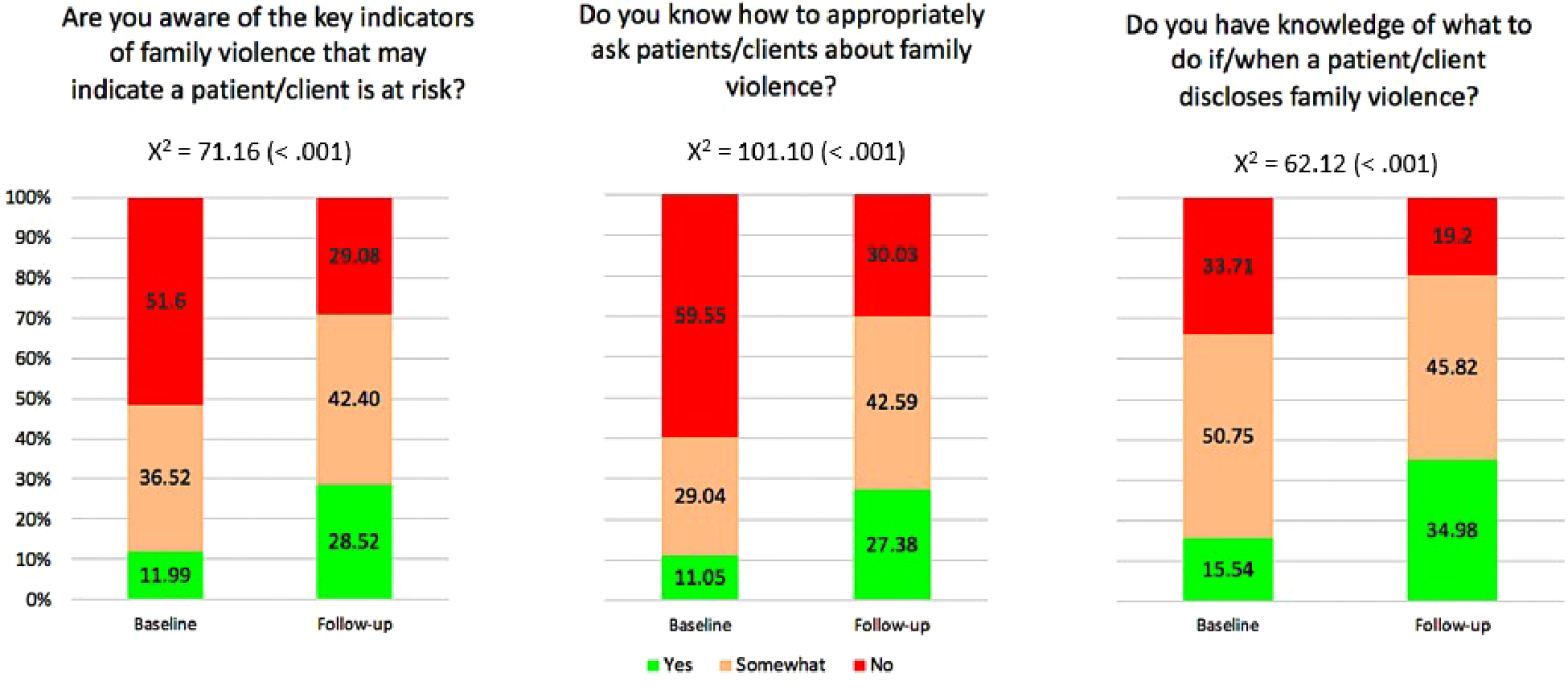
Clinician self-ratings of family violence key indicators, enquiry skills, and managing disclosures.

Consistent with the knowledge and confidence results, a similar trajectory of improvement was observed between the baseline and 3-year follow-up time points, in the areas of specific family violence response skills. Results are graphically represented in Figure 1. On *χ*^2^ comparisons, significantly more clinicians responded *Yes*, or *Somewhat* to questions about knowledge of key family violence indicators, how to ask patients about family violence, and how to respond to disclosures, at follow-up, compared to baseline. These differences remained significant after Bonferroni correction.

The final section of the survey relates to barriers that impact on the capacity of clinicians to address patient family violence issues (see Table 4). As an overall trend, fewer barriers were identified by clinicians, with all 13 pre-specified barriers showing a reduction in endorsement at the follow-up time point. The barriers with the most notable decreases in endorsement were lack of clinician knowledge for asking, concerns about rapport, lack of policies/procedures, impact on staff safety, and access to supervision/reflective practice. These five areas showed significant changes in levels of endorsement across the two time points.

**Table 4:** Challenges in addressing family violence endorsed by respondents.

Barrier	Baseline %	Follow-up %	Test statistic (*p* value)
The patient/client's partner/child/parent (i.e. suspected perpetrator) is present	58.41^a^	48.67^a^	*Χ*^2^ = 0.6.61 (0.010)
Patient/client's reluctance to disclose when asked	54.16^a^	48.29^a^	*Χ*^2^ = 1.82 (0.18)
Time limitations when seeing a patient/client	51.54^a^	46.58^a^	*Χ*^2^ = 0.87 (0.35)
I don't know what to do or say	48.74	27.76	*Χ*^2^ = 42.68 (<0.001)
Language barriers	46.81	41.63	*Χ*^2^ = 1.46 (0.23)
Concern about offending the patient/client or affecting rapport	44.29	33.27	*Χ*^2^ = 10.38 (0.001)
Lack of supporting policies and procedures	35.59	9.89∼	*Χ*^2^ = 92.44 (<0.001)
Privacy issues in the clinical area in which I work	35.20	28.52	*Χ*^2^ = 3.82 (0.051)
Another vulnerable person is present (i.e. children)	34.62	25.86	*Χ*^2^ = 7.45 (0.006)
The topic of family violence is uncomfortable	25.53∼	17.68	*Χ*^2^ = 7.81 (0.005)
Concerns about staff safety in asking questions about family violence and initiating action	25.34∼	14.26∼	*Χ*^2^ = 17.86 (<0.001)
Little or no access to supervision that supports safe and reflective practice in this area	23.79∼	11.41∼	*Χ*^2^ = 25.08 (<0.001)
Other (please specify)	6.19	3.61	*Χ*^2^ = 3.29 (0.070)

∼ Three least commonly endorsed barriers.

^a^ Three most commonly endorsed barriers.

The three most commonly endorsed pre-specified barriers remained the same across time points. There was some change in the least commonly endorsed barriers, however, with *Lack of supporting policies and procedures* the least commonly endorsed barrier at follow-up, a change from baseline. This was also the pre-specified barrier that showed the highest magnitude of change, across the two time points.

Further analysis of the follow-up data set is being conducted, including an analysis stratifying the respondents into groups according to the amount of family violence training they had received (i.e., none, some, Family Safety Advocate/clinical champion training) for both the quantitative and qualitative data portions of the survey. To generalize, at a very broad level, results from the qualitative analysis of the text box response data indicates that staff trained in the clinical champions program show a greater depth of knowledge, and skills that are more aligned with best-practice guidelines, relative to staff with short-duration, or not training. However, staff with shorter-duration training still generally demonstrate stronger family violence clinician response knowledge than those with no training. This analysis will be presented in subsequent papers.

## Discussion

The data presented in this paper indicates that overall, the SHRFV initiative and the transformational change project at the Royal Melbourne Hospital was effective in improving family violence knowledge and skills in clinical staff. Whilst training was a large component of the initiative, the project went beyond training and encompassed policy implementation, clinical workflow, a secondary consultation service, awareness raising, and a clinical champions' model including a community of practice. In most areas, statistically significant improvements in self-rated family violence skills proficiency were observed. This provided promising evidence that a large health service, starting from a low knowledge base, can make meaningful gains in clinician family violence knowledge with appropriate funding, skills and resources, and a clear project plan and direction.

Importantly, at 3-year follow-up, the clinician cohort self-ratings indicated significantly greater knowledge about family violence relative to the baseline cohort, and the screening of patients more frequently. Clinician confidence working in the area also improved from baseline – but continued to lag behind self-rated knowledge levels. This suggests that even with increased training and knowledge clinicians may not be confident in their capacity to apply these skills clinically when working with patients. Improvement in clinicians endorsing the frequency with which they work with clients experiencing family violence did not reach statistical significance. At a surface level, this may suggest that clinicians are still not recognising that family violence may be occurring for their clients, and thus not screening when it is indicated. However, it could be that clinicians are interpreting this question to refer to patients who come to see them with a known and documented history of family violence, prior to any screening conducted by the individual clinician, themself. Future research, *via* auditing the uptake use of the family violence screening tool in the electronic medical record, will assist to evaluate actual clinician behaviour with screening.

At 3-year follow-up Allied Health staff tended to rate their family violence skills more strongly than Nursing or Medical staff. This is likely to reflect the fact that the majority of the Family Safety Advocates (clinical champions) who had received more in-depth training, came from Allied Health. Further the professions of Social Work and Psychology sit within this professional grouping, and all staff in these teams were required to undertake the advocate training as part of their job role.

Encouragingly, clinician self-ratings of specific family violence skills improved in all three assessed areas. This included knowledge of key indicators, asking about family violence, and responding to disclosures. The data on clinician-identified barriers to working effectively in the area of family violence also yielded interesting results. Notably, there was a large and significant decrease in staff identifying a lack of supporting policies and procedures to do this work and far fewer staff endorsed the barrier of not knowing what to do or say, at follow-up. This is likely to reflect the fact that the Royal Melbourne Hospital had established a family violence policy and response procedure to guide clinical staff practice at the time of follow-up; something that had not existed at baseline. It also suggests that both the awareness raising and training in the response procedure were beneficial to improve staff knowledge with both asking and responding.

Notable also, the three most highly endorsed barriers to working effectively in the area remained the same across time points. A suspected perpetrator being present during the clinical interaction is a safety risk that impacts on the capacity of staff to enquire safety with victim/survivors. Family violence training at the hospital has included brainstorming about how to separate suspected victim/survivors and suspected perpetrators, so that safe family violence enquiry can occur. However, data from the current study suggests further work could be done in this area. Similarly, nearly 50% of clinicians continue to believe that clients will be reluctant to disclose when asked. Thus, future training should continue to reinforce that multiple occasions of asking may be required, and that many victim/survivors want to be asked and provided with support (22, 26, 32). The issue of time limitations also continued to be raised by busy clinicians who struggle to fit in family violence screening with other routine care procedures. Reinforcement with staff that family violence impacts significantly on physical and mental health may assist with further reducing this barrier, as well as increased effort by the Royal Melbourne Hospital to make identifying signs and screening a required part of routine clinical care.

While the overall results of this research are promising, they also indicate that further and ongoing resourcing is needed in this area. Twenty-five percent of the clinician respondents at follow-up indicated that they had never undertaken any training in family violence. This is far from optimal, as it suggests that one-quarter of the clinical staff patients encounter have had no training in this area. Thus, many staff may miss key indicators that patients are experiencing family violence, and/or responding inadequately, or inappropriately, if patients choose to disclose family violence issues to them. Further, sizeable proportions of staff still rate their knowledge and confidence working clinically in the area of family violence as low, and respond with a definitive *No*, when asked if they have an understanding of specific family violence clinical skills (indicators, asking and responding). Thus, despite the progress made over three-years at the Royal Melbourne Hospital, further and ongoing work is needed to optimise staff knowledge levels.

The local environment of the hospital at the time of the follow-up study should also be considered. In contrast to baseline, the follow-up was conducted in the midst of the COVID-19 pandemic. Data collection was undertaken several weeks after a four-month, government-imposed lock-down. The hospital at which this study was conducted was the most heavily impacted by COVID-19 in the country in 2020, with the highest number of patient deaths and staff infections, affecting staff wellbeing (33, 34). This impacted on the capacity of the Family Safety Team to continue with the family violence training schedule, with a reduction in the provision of training and fewer attendances at training, compared to the previous calendar year (4,309 attendances at training in 2019; 1,089 in 2020; (29). Members of the team were also redeployed to acute clinical and staff COVID-19 support roles during the COVID-19 surges, with some converted to work-from-home arrangements to minimise infection in non-frontline staff. The follow-up survey was administered at a time when the clinical workforce was impacted by fatigue and burnout from the COVID-19 surge. This may have affected engagement levels. It was anticipated by the study team that participation would be higher at follow-up, relative to baseline, due to the levels of awareness raising in the area over the previous three years. In contrast to this, a small drop in participation numbers was seen.

Limitations to this study also include the overall survey response rate in the follow-up cohort of 17.31%. This response rate is not optimal for confidently generalising the results to the broader clinical staff cohort. However, it is commensurate with the baseline survey response rate, and with other online only healthcare worker surveys administered *via* email contact only, with large (>2,000) participant contact pools (35, 36). The response rate to the survey is also far greater than several other recent family violence knowledge surveys in healthcare worker cohorts (37, 38).

The majority of the training provided fit within a cognitivism based instrumental learning framework. However, the lengthier clinical champions training (Family Safety Advocates) also included transformative and social learning principles, and participants were required participants to undertake experiential exercises. Overall, this study cannot be considered a direct assessment of any individual family violence training type, module, or program, as many different types were employed during the course of the Family Safety Team SHRFV initiative at the Royal Melbourne Hospital, including some sourced through external providers. Rather, it is reflective of the impact of the entire multi-faceted transformational change project at the service. However, it does sit within the broader context of evaluating staff training in family violence. Previous studies have indicated that healthcare worker family violence clinical training seminars of 1–2 days duration have some effect in improving knowledge of family violence skills, attitudes to screening, service culture and patient satisfaction, but that these may have small effect sizes and may not translate to any increase in the identification of family violence in the patient cohort (16, 17, 39). Sustained and comprehensive initiatives, where options for repeated and in-depth training are available, in services with wrap-around policies and procedures in family violence, may be more effective at improving family violence knowledge and practices, albeit more costly and labour intensive.

What remains to be determined at the Royal Melbourne Hospital is whether there has been direct and measurable improvement in care for patients with current family violence situations attending the service. A repeat of the baseline patient survey has been hampered due to the fluctuating and ongoing COVID-19 situation, limiting the majority of non-essential research from face-to-face settings. However, a systematic audit of the uptake and utilisation of the family violence screening and clinical workflow is planned, as well as an evaluation of use of family violence alerts placed on patient files by clinicians. Further research in progress at the service includes a psychometric study of the test-retest validity of the clinician survey tool to supplement the internal consistency evaluations that have already been undertaken.

Family violence remains a significant problem that impacts on the health and wellbeing of victim-survivors and conveys a high economic burden (40, 41). The role of healthcare systems in identifying and supporting people experiencing family violence is well recognised in many countries; although healthcare workers remain under educated and supported to do this work at best-practice standards. The results of the current study suggest that measurable improvement can be made in healthcare worker family violence clinical skills knowledge, when a comprehensive, transformational change project is implemented in a large relatively well resourced, adult hospital. To achieve this, comprehensive service changes and supports are required, in addition to wide-scale training. Findings underscore the need for ongoing resourcing in family violence training and supporting structures, such as secondary consultation and a community of practice, to assist clinicians to continue to provide an effective family violence response to patients.

## Data Availability

The raw data supporting the conclusions of this article will be made available by the authors, without undue reservation.
